# Comparative plastid genomics of Synurophyceae: inverted repeat dynamics and gene content variation

**DOI:** 10.1186/s12862-018-1316-9

**Published:** 2019-01-11

**Authors:** Jong Im Kim, Hyunmoon Shin, Pavel Škaloud, Jaehee Jung, Hwan Su Yoon, John M. Archibald, Woongghi Shin

**Affiliations:** 1 Department of Biology, Chungnam National University, Daejeon, 34134 South Korea; 2 Department of Botany, Faculty of Science, Charles University, Benátská 2, CZ-12800 Prague 2, Czech Republic; 3 Department of General Education, Hongik University, Seoul, 04066 South Korea; 4 Department of Biological Sciences, Sungkyunkwan University, Suwon, 16419 South Korea; 5 Department of Biochemistry and Molecular Biology, Dalhousie University, Halifax, Nova Scotia B3H 4R2 Canada

**Keywords:** Algae, Stramenopiles, Synurophyceae, Plastid genomes, Lateral gene transfer

## Abstract

**Background:**

The Synurophyceae is one of most important photosynthetic stramenopile algal lineages in freshwater ecosystems. They are characterized by siliceous scales covering the cell or colony surface and possess plastids of red-algal secondary or tertiary endosymbiotic origin. Despite their ecological and evolutionary significance, the relationships amongst extant Synurophyceae are unclear, as is their relationship to most other stramenopiles.

**Results:**

Here we report a comparative analysis of plastid genomes sequenced from five representative synurophycean algae. Most of these plastid genomes are highly conserved with respect to genome structure and coding capacity, with the exception of gene re-arrangements and partial duplications at the boundary of the inverted repeat and single-copy regions. Several lineage-specific gene loss/gain events and intron insertions were detected (e.g., *cem*A, *dna*B, *syf*B, and *trn*L).

**Conclusions:**

Unexpectedly, the *cem*A gene of Synurophyceae shows a strong relationship with sequences from members of the green-algal lineage, suggesting the occurrence of a lateral gene transfer event. Using a molecular clock approach based on silica fossil record data, we infer the timing of genome re-arrangement and gene gain/loss events in the plastid genomes of Synurophyceae.

**Electronic supplementary material:**

The online version of this article (10.1186/s12862-018-1316-9) contains supplementary material, which is available to authorized users.

## Introduction

The Synurophyceae, a class of photosynthetic stramenopile (or heterokont) algae, is a morphologically diverse lineage with plastids derived from red algae via secondary or tertiary endosymbiosis. They are motile organisms with two parallel emergent flagella, one or two plastids, a cell coat in which the siliceous scales cover the entire cell, and lack of an eyespot [1, 2]. Synurophyceae are presently assigned to one of three genera: *Mallomonas*, *Neotessella* and *Synura*. Members of the genus *Synura* are colonial flagellates characterized by cells having two visible and unequal flagella, two plastids, and an external covering of siliceous scales. Members of the single celled genus *Mallomonas* have silica scales and bristles, while the colonial genus *Neotessella* is characterized by an oval-shaped scale structure and a single scale case that surrounds the whole colony.

The presence of four membranes surrounding Synurophyceaen plastids provides direct evidence for the hypothesis that their plastids are derived by eukaryote-eukaryote endosymbiosis, a process that is thought to have given rise to photosynthesis in several other protist lineages (e.g., cryptophytes, haptophytes, euglenoids, chlorarachniophytes; [3–5]). The outermost membrane of synurophycean plastids is continuous with the endoplasmic reticulum (referred to as the chloroplast ER; CER), but unlike the red-algal derived plastids of other ‘chromists’ such as haptophytes and cryptophytes, a linkage between this membrane and the outer nuclear envelope is either totally lacking or marginal [1]. The silica deposition vesicles (SDVs), in which siliceous scales form, are produced from the CER on the outer side of the plastid. The mature scales are brought to the cell surface via Golgi body vesicles and placed in position alongside pre-existing scales [6, 7].

Molecular sequence datasets that include combinations of nuclear, plastid, and mitochondrial genes have provided insight into the branching order amongst the three recognized synurophyte genera and their phylogenetic relationship to other algae [8–10]. Recently, the plastid genome of the chrysophycean alga *Ochromonas* sp. CCMP1393 was reported [5]. The *Ochromonas* species genome was ‘conservative’ in possessing a large single copy region (LSC), a very short single copy region (SSC), and two inverted repeats (IR) with 15 functional protein-coding genes and ribosomal RNA operons. Recent phylogenomic studies of photosynthetic stramenopiles based on plastid genome data have focused mainly on Bacillariophyceae (diatoms) and Phaeophyceae (brown algae) with one or a few species from six additional classes—Bolidophyceae, Chrysophyceae, Eustigmatophyceae, Pelagophyceae, Raphidophyceae, Xanthophyceae—as well as plastid-bearing alveolates [5, 11]. While photosynthetic stramenopiles consist of at least 15 classes, phylogenetic relationships amongst them, including Synurophyceae, are still unresolved. Investigation of organellar genome structure and coding capacity from new protists has the potential to complement phylogenetic analyses by reinforcing observed relationships and helping to resolve phylogenetic issues.

Lateral gene transfer (LGT; also known as horizontal gene transfer) is the movement of genetic material from one species into the genome of an unrelated species. LGT provides a potentially important source of genetic variation in mitochondrial and plastid genomes, many of which display intron gain/loss and the presence of chimeric genes created by gene conversion. LGT appears to be rare in algal plastid genome but a few probable cases of bacterial derived genes have nevertheless been documented. These include the leucine biosynthesis (*leu*C/D) operon and RuBisCO genes of red algae [4, 12], the *dna*X gene and group II introns in cryptophytes [13–16], the *rpl*36 gene in the haptophyte and cryptophyte plastid genomes [13] and the *ebo* operon in Eustigmatophyceae [17]. In addition, the plastid genomes of several angiosperms show evidence for LGT of one or more genes from mitochondria to plastids [18–20]. Although in most cases the underlying mechanisms are not known, taken together these examples strongly suggest that LGT from bacteria to organelles and from one organelle to another can occur.

Here, we present five complete plastid genome sequences from the three morphologically distinct genera of synurophycean algae: *Mallomonas, Neotessella,* and *Synura*. To better understand the relationships among synurophytes as well as the broader insight into organellar genome evolution of plastids in stramenopiles, we performed comparative and phylogenetic analyses of these genomes in the context of publicly available plastid genome sequence data, including that of the chrysophycean alga, *Ochromonas* sp. CCMP1393. Our results reveal highly conserved features of plastid genomes amongst the synurophyceans. We also uncovered several examples of gene loss/gain, duplication and gene rearrangement. Our results provides important insights into the evolutionary history of organelle genomes via lateral gene transfer (LGT) from green-algal lineages into the Synurophyceae, as well as divergence time estimates using molecular clock approaches based on silica fossil records. Collectively, our data contribute to a better understanding of the evolutionary history of the Synurophyceae.

## Results

### General features of Synurophyceae plastid genomes

**Fig. 1:**
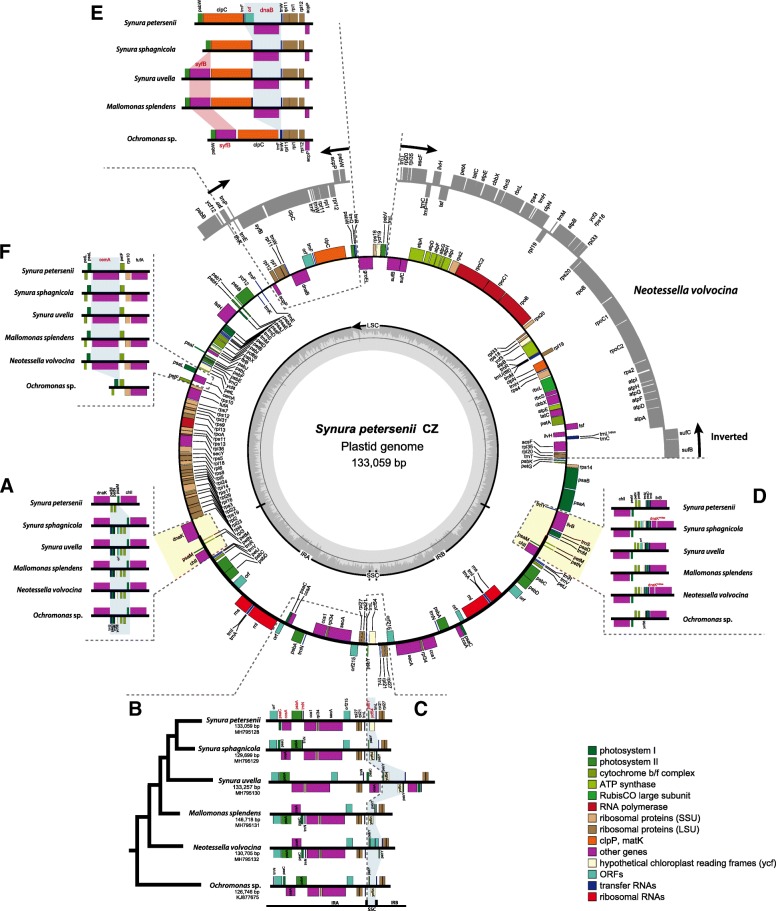
Circular map of the plastid genome of *Synura petersenii*. The gene content and arrangement of the synurophycean plastid genomes examined herein are identical, with the exception of the six syntenic regions shown as (**a**-**f**). Regions of the *N. volvocina* genome are shown in gray. The protein coding genes, rRNA and tRNA genes are labeled inside or outside of the circle. The genes are color-coded according to the functional categories in the index

**Fig. 2:**
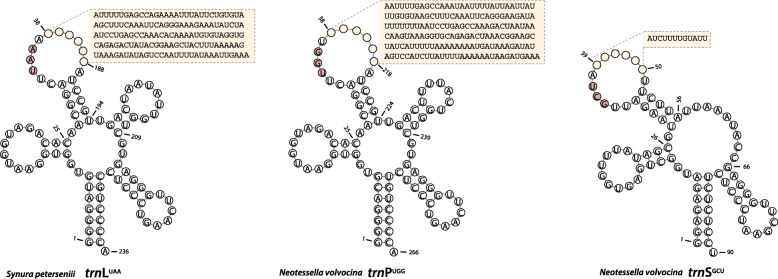
tRNAs structures with intron sequences in synurophycean plastid genomes. Collectively, these tRNAs show outright gene losses, pseudogenization, and intron insertions. The nucleotides in red indicate anti-codon

Five new plastid genomes (ptDNA) were sequenced from the synurophycean genera *Mallomonas*, *Neotessella*, and *Synura* (Table 1, Fig. 1). The structure and coding capacity of these ptDNAs were then compared to the published genome of the related chrysophycean alga, *Ochromonas* sp. CCMP1393 [5]. The plastid genome sizes of the Synurophyceae ranged from ~ 130 kbp (*S. sphagnicola*) to ~ 147 kbp (*M. splendens*) and the overall GC content ranged from 37.5 to 42.4%. The overall organization of the five synurophytes and *Ochromonas* sp. CCMP1393 was found to be conserved: they each contain a large single copy region, a very short single copy region, and two inverted repeats. The plastid genomes of Synurophyceae share a core set of 134 functional protein-coding genes including genes in the IR regions (Table 2). The plastid genome IR sequence length of the Synurophyceae and *Ochromonas* sp. CCMP1393 ranged from 22.5 kbp to 31.6 kbp with 15 functional protein-coding genes (*ccs*1, *ccs*A, *chl*I, *pet*J, *pet*M, *pet*N, *psa*C, *psa*M, *psb*A, *psb*C, *psb*D, *rpl*21, *rpl*27, *rpl*34, and *sec*A), 3 rRNAs and 5 tRNAs. Introns and a pseudogene were found in the tRNA genes. The *trn*R^UCU^ in *S. petersenii*, *S. uvella*, and *M. splendens* and *trn*E^UUC^ in *N. volvocina* is present as a pseudogene; it has a low hidden Markov model score (HMM score = 0) and a secondary structure-only score (2Str Score < 40) predicted by tRNAscan-SE. *Synura* and *Mallomonas* have an intron in *trn*L^UAA^, whereas *N. volvocina* has introns in *trn*P^UGG^ and *trn*S^GCU^ (Table 3, Fig. 2). The five newly determined plastid genomes showed a high degree of structural conservation relative to the representative species, *Synura petersenii* (Fig. 1 and Additional file 1: Figure S2). Gene order among Synurophyceae and *Ochromonas* ptDNAs was conserved, with the exception of *N. volvocina*, which has two inversions that differ from other synurophycean species (Fig. 1).

**Table 1:** Characteristics of Synurophyceae plastid genomes analyzed in this study

General characteristics	*Synura petersenii* S114.C7, CZ	*Synura sphagnicola* FBCC200022	*Synura uvella* FBCC200023	*Mallomonas splendens* CCMP1872	*Neotessella volvocina* CCMP1871	*Ochromonas* species CCMP1393
Key characteristics for genus classification	colonized cells covered with silica scale on each cell	colonized cells covered with silica scale on each cell	colonized cells covered with silica scale on each cell	single cell covered with silica scale	colonized cells covered with silica scale on colony	naked single cell
Size (bp)	133,059	129,699	133,257	146,918	130,705	126,746
Inverted repeat (IR)	23,151	22,505	23,691	31,611	24,064	22,906
Small single-copy region	1,135	1,191	2,939	711	2,432	805
Large single-copy region	85,622	83,498	82,936	82,985	80,145	80,129
G+C (%)	37.89	38.76	38.19	42.39	37.54	30.9
Total gene (include RNAs)	182	181	189	187	186	183
No. of protein-coding genes	144	144	151	150	149	144
tRNAs	34	33	34	33	33	33
rRNA operons	2	2	2	2	2	2
Introns	*trn*L	*trn*L	*trn*L	*trn*L	*trn*P, *trn*S	-
Unknown ORFs	8	3	9	11	9	7
pseudogene	-	-	*trn*R	*trn*R	*trn*E	-
partial copied gene	-	*dna*K	-	*-*	*dna*K	*-*
specific encoded genes	*dna*B/ *cem*A	*dna*B/ *cem*A	*dna*B/ *cem*A	*dna*B/ *cem*A	*cem*A	-
missing gene	*syf*B	*syf*B	-	-	-	-
GenBank accession	MH795128	MH795129	MH795130	MH795131	MH795132	KJ877675

**Table 2:** List of genes in the synurophycean plastid genome

Classification	Genes							
Genetic systems								
Maintenance	dnaB^S,M^							
RNA polymerase	rpoA	rpoB	rpoC1	rpoC2				
Translation	tsf	tufA	syfB^Su,M,N^					
Protein quality control	clpC	clpN	dnaK	ftsH	groEL			
Transport								
Transport	cemA^S,M, N^	secA^2^	secY	sufB	sufC	tatC		
ATP synthesis								
ATP synthase	atpA	atpB	atpD	atpE	atpF	atpG	atpH	atpI
Ribosomal proteins								
Large subunit	rpl1	rpl2	rpl3	rpl4	rpl5	rpl6	rpl11	rpl12
	rpl13	rpl14	rpl16	rpl18	rpl19	rpl20	rpl21^2^	rpl22
	rpl23	rpl24	rpl27^2^	rpl29	rpl31	rpl33	rpl34^2^	rpl35
	rpl36							
Small subunit	rps2	rps3	rps4	rps5	rps7	rps8	rps9	rps10
	rps11	rps12	rps13	rps14	rps16	rps17	rps18	rps19
	rps20							
Metabolism								
Carbohydrates	rbcL	rbcS						
Lipids	acpP							
Nucleotides								
Amino acids	ilvB	ilvH						
Cofactors	ascF	chlI^2^						
Photosystems								
Photosystems I	psaA	psaB	psaC^2^	psaD^2^	psaE	psaF	psaI	psaJ
	psaL	psaM^2^	ycf3	ycf4				
Photosystmens II	psbA^2^	psbB	psbC^2^	psbD^2^	psbE	psbF	psbH	psbI
	psbJ	psbK	psbL	psbN	psbT	psbV	psbW	psbX
	psbY^2^	ycf12						
Cytochrome complex	cbbX	ccs1^2^	ccsA^2^	petA	petB	petD	petF	petG
	petJ^2^	petL	petM^2^	petN^2^				
Redox system	ftrB							
Unknown								
Conserved ORFs	orf215^2^	ycf19	ycf36^2^	ycf54	ycf66			

Note. ^2^present as repeated genes, ^M^present in *Mallomonas splendens*, ^N^present in *Neotessella volovocina*, ^S^present in three species of genus *Synura*, ^Su^present only in *Synura uvella* in plastid genome

**Table 3:** tRNAs present in Synurophyceae plastid genomes.

	*Synura petersenii* S114.C7	*Synura sphagnicola* FBCC200022	*Synura* *uvella* FBCC200023	*Mallomonas splendens* CCMP1782	*Neotessella volvocina* CCMP1781	*Ochromonas* sp. CCMP1393
trnA(UGC)^2^	2	2	2	2	2	2
trnC(GCA)	1	1	1	1	1	1
trnD(GUC)	1	1	1	1	1	1
trnE(UUC)	1	1	1	-	1^Ψ^	1
trnF(GAA)	1	1	1	1	1	1
trnG(UCC)	1	1	1	1	1	1
trnH(GUG)	1	1	1	1	1	1
trnI(GAU)^2^	2	2	2	2	2	2
trnK(UUU)	1	1	1	1	1	1
trnL(UAA)	1^I^	1^I^	1^I^	1^I^	-	1
trnL(CAA)	1	-	1	1	-	-
trnL(UAG)^2^	2	2	2	2	2	2
trnM(CAU)^2^	3	4	4	4	4	3
trnfM(CAU)	-	-	-	-	-	1
trnN(GUU)^2^	2	2	2	2	2	2
trnP(UGG)	1	1	1	1	1+1^I^	1
trnQ(UUG)	1	1	1	1	1	1
trnR(ACG)	1	1	1	1	1	1
trnR(CCG)	1	-	-	-	-	-
trnR(UCU)^2^	2 ^Ψ^	2	2 ^Ψ^	2 ^Ψ^	2	2
trnS(GCU)	-	1	1	1	1^I^	1
trnS(UGA)^2^	2	2	2	2	2	2
trnT(UGU)	1	1	1	1	1	1
trnU(UUUU)	1	-	-	-	-	-
trnV(UAC)^2^	2	2	2	2	2	2
trnW(CCA)	1	1	1	1	1	1
trnY(GUA)	1	1	1	1	1	1
Total	34	33	34	33	33	33

Note. ^2^present as repeated tRNA genes, ^I^present as intron encoded tRNA genes, ^Ψ^present as tRNA pseudogene in plastid genome

### Gene arrangements of IR and SSC regions

The synurophycean plastid genomes exhibit different gene order patterns in six distinct regions (Fig. 1a-e). First, three different gene order patterns were found at the boundaries between single-copy and repetitive regions (Fig. 1a). The most common pattern is that shared by *Ochromonas* sp. CCMP1393, *N. volvocina* and *M. splendens,* in which a particular block of genes (*trn*S-*psa*D-*trn*M-*ycf*36-*pet*M-*pet*N) lies between the *dna*K and *chl*I genes at the IRA/LSC junction (Fig. 1a). Within this syntenic block, *S. uvella* and *S. sphagnicola* have one open reading frame (ORF) between *ycf*36 and *pet*M, whereas *S. petersenii* is distinct in the loss of *trn*S-*psa*D-*trn*M-*ycf*36 (Fig. 1a). Second, four different gene order patterns were observed at the IR/SSC junction (Fig. 1b). Gene content in this region of the genome is conserved, but four genes (*psa*C, *ccs*A, *psb*A, *trn*N) are dynamically rearranged in the IR regions of each species. The gene rearrangements in the plastid genome of *S. uvella* are distinct from other synurophyceans. The IRB/LSC junctions of the synurophyte plastid genomes also showed three different gene order patterns (Fig. 1d). The first pattern, shared by *Ochromonas* sp. CCMP1393, *M. splendens* and *S. petersenii,* involves the *ilv*B gene linked to the *trn*S-*psa*D-*trn*M genes. It probably represents the ancestral gene order in the synurophycean algae. The second pattern is seen only in *S. uvella*, one in which there is an ORF in the plus orientation between *ycf*36 and *pet*M. The third pattern is shared by *N. volvocina* and *S. sphagnicola*; it involves the presence of a partial *dna*K gene (the duplicated region starts at the 440th amino acid) between the *ilv*B and *trn*S-*psa*D-*trn*M genes.

The small single copy (SSC) region is exceptionally short, ranging from 711 bp to 2939 bp, and includes only two protein-coding genes: *ycf*54 and *psb*Y (Fig. 1c). The SSC flanking regions have slightly different patterns of gene duplication and location of the genes in each species. The *psb*Y gene is located to the left side of *ycf*54 gene in *S. petersenii* and *Ochromonas* sp. CCMP1393, while it is located on the right side in *M. splendens*. *Synura sphagnicola* has duplicated *psb*Y genes on both sides of *ycf*54, but the *ycf*54 gene is absent in plastid genome of *N. volvocina*. *Synura uvella* has duplicated *psb*Y-*ycf*54 genes in extended IR regions.

## Discussion

### Expansions and contractions of IR region

The expansions and contractions of IR region have occurred frequently during the evolutionary history of Synurophyceae, leading not to changes in gene content, but to gene rearrangements and gene duplications in our results. Such events can alter gene order through inversion, expansion/contraction of the IR, gene duplication/loss, or transposition. IR boundary shifts are a common phenomenon, which is thought to be caused by inversions or recombination between repeated sequences resulting in gene order changes in plastid genomes [21, 22]. Contractions, expansions and small-scale changes in IR and SSC regions appear to be common in diatoms and green algae, leading to dynamic gene rearrangements and changes in gene content [23–26]. Rearrangements at the IR boundary is likely one of the factors contributing to the extensive genome rearrangements in the Synurophyceae as well.

### Lineage specific gene gain and loss

Previous work has shown that in red-algal derived secondary plastids, most of the lineage-specific plastid genes show complex distribution patterns suggesting independent losses across a broad range of phylogenetic depths [16]. Although the plastid genomes of Synurophyceae and *Ochromonas* sp. CCMP1393 studied herein are generally highly conserved in structure and gene content, three genes were identified as being lineage specific: *dna*B, *syf*B, and *cem*A (Fig. 1e-f). To understand the evolutionary distribution and phylogenetic relationships of these genes among eukaryotes, we performed phylogenetic analyses of homologs obtained from the plastid genomes of major photosynthetic eukaryotic groups with their cyanobacterial homologs.

The *dna*B gene encodes a DNA helicase that is involved in organelle division [27, 28] and is found in the plastid genome of cryptophytes, some dinoflagellates (i.e., those with diatom-derived plastids), and specific subgroups of stramenopiles and rhodophytes (Additional file 2: Figure S3; [16]). Interestingly, *dna*B in Synurophyceae was only found in the genera *Synura* and *Mallomonas* (Fig. 1e and Additional file 2: Figure S3). The synurophyte sequences branch at the base of the photosynthetic stramenopiles in the algal *dna*B gene tree (Additional file 2: Figure S3). In stramenopiles, *dna*B is present only in Bacillariophyceae (except *Synedra acus*), Phaeophyceae, Raphidophyceae, *Triparma,* Synurophyceae and Xanthophyceae, but absent in Pelagophyceae, Eustigmatophyceae, and Chrysophyceae [16]. Of particular note, the ‘dinotoms’ *Durinskia baltica* and *Krptoperidinium foliaceum*, which are dinoflagellates harboring a diatom endosymbiont, contain a *dna*B gene in their plastid genomes [29]. The cryptophytes, which also harbor a complex red-algal derived plastid, branch with the main red algal lineage including *Galdieria sulphuraria*. The *dna*B gene appears to have been present in the plastid genome of the red algal common ancestor; if it was present in the common ancestor of all primary plastid-bearing algae, it was lost in green algae and glaucophytes, and independently in many complex red-algal derived plastid genomes (Additional file 2: Figure S3).

The *syf*B gene encodes the β subunit of phenylalanyl-tRNA synthetase [30]. While the *syf*B and *syf*H genes are retained in primary plastid-bearing organisms, the *syf*H gene is absent in complex red-algal plastid genomes. Furthermore, *syf*B has been lost in almost all red algl-derived plastid genomes, with the exception of the diatom *Triparma* and Chrysophyceae in stramenopiles [5, 11, 24, 31], as well as rhodophytes [32]. In the Synurophyceae and Chrysophyceae, *syf*B remains in most species, but is not present in *S. petersenii* and *S. sphagnicola*, suggesting that it was lost recently in a common ancestor shared by these two species (Fig. 1e, and Additional file 3: Figure S4). The dinotoms *Durinski baltica* and *Krptoperidinium foliaceum* also have a *syf*B gene in their plastid genomes. It is likely that the *syf*B gene has a similar history as *dna*B, i.e., being ancestrally present and lost independently in specific groups.

### Lateral gene transfer from green algae into photosynthetic Stramenopiles

**Fig. 3:**
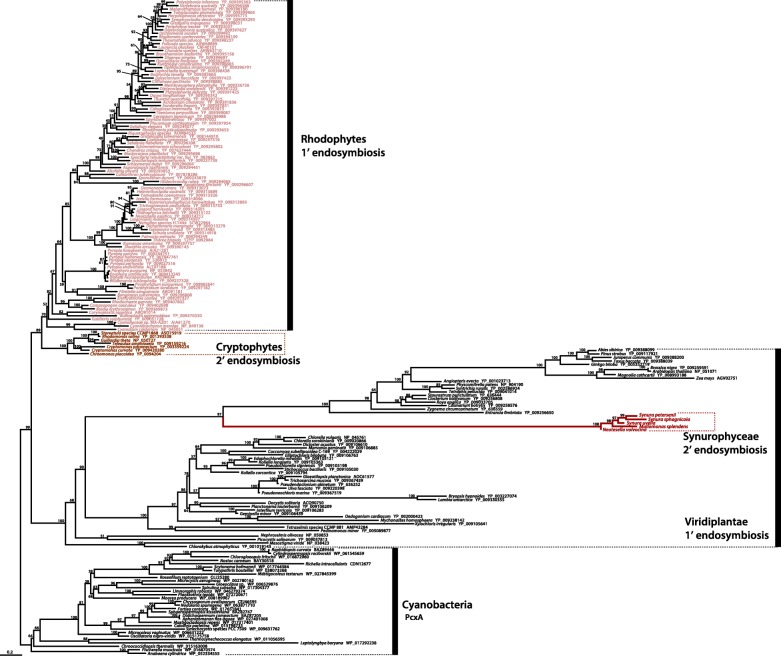
Phylogenetic tree based on *cem*A protein sequences. Numbers on branches are IQ-Tree UFBoot values. The scale bar shows the inferred number of amino acid substitutions per site

The *cem*A gene encodes a chloroplast inner membrane protein [33]; it is conserved in almost all green algae, liverworts, land plants and rhodophytes, but is not found in glaucophytes [34, 35]. In the green algal order Bryopsidales, the *cem*A gene appears to have been lost in two clades [36]. Among the groups of algae with red-algal derived complex plastids, the *cem*A gene is thus far only found in cryptophytes and Synurophyceae (Figs. 1f and 3), and our phylogenetic analyses suggest that the *cem*A homologs in these two lineages have different origins: the cryptophyte protein shows a strong phylogenetic relationship with rhodophytes, whereas the synurophycean protein groups within Viridiplantae (Fig. 3). At face value, this is consistent with the hypothesis that the synurophycean plastid *cem*A gene was derived from a member of the green lineage through LGT. It is, however, not possible to be more specific than this; the synurophycean homologs are extremely divergent and branch sister to long-branching *cem*A proteins in streptophytes, rather than those of chlorophytes.

### Evolution of the *trn*L^UAA^ intron

The *trn*L^UAA^ group I intron of algae is thought to have been acquired from the ancestral cyanobacterial endosymbiont that gave rise to the plastid. The existence of related introns in the *trn*L^UAA^ gene has been reported in most green algal plastid genomes, as well as some stramenopiles [37]. A phylogenetic analysis of the intron suggests that it was present in the cyanobacterial ancestor of the three primary plastid-bearing lineages i.e., Rhodophyta, Viridiplantae, and Glaucophyta (Additional file 4: Figure S5). The *trn*L^UAA^ group I intron is absent from red, cryptophyte and haptophyte algae, and found only in some stramenopiles, i.e., Phaeophyceae, Phaeothamniophyceae, Xanthophyceae, and Eustigmatophyceae ([5, 38, 39], this study). Given the high degree of intron sequence similarity between these four subgroups of stramenopiles, the *trn*L^UAA^ gene is probably derived from the same ancestral archaeplastidal sequence. One notable feature is the presence of a predicted group I intron in all *trn*L^UAA^ and *trn*P^UGG^ (*Neotessella volvocina*) genes in the synurophycean plastid genome (Fig. 2). The group I intron sequences are more closely related to homologs in the green algal lineage and chlorarachniophytes (which have a green algal secondary plastid), rather than other stramenopiles (Additional file 4: Figure S5). However, it is not possible to infer the origin *trn*L intron with certainty, as the structure of the *trn*L^UAA^ group I intron tree is generally very poorly supported.

### Evolutionary history of plastid genomes in Synurophycean algae

**Fig. 4:**
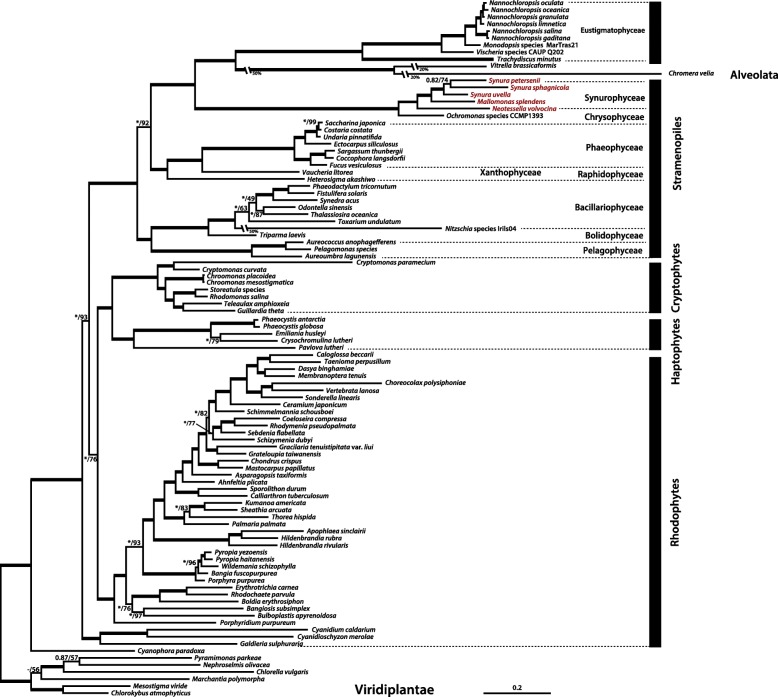
Phylogenetic tree of synurophyte plastids. This tree was constructed using a dataset of 91 concatenated proteins (18,250 amino acids). The numbers on each node represent ultrafast bootstrap approximation (UFBoot) values (left) calculated using IQ-Tree and posterior probabilities (right). The bold branch indicates strong sipported values (ML = 100 / PP = 1.00). The scale bar indicates the number of substitutions/site

Phylogenomic analysis using 91 genes of plastid genome data showed a monophyletic, strongly supported (MLB = 100%) synurophycean clade; internal relationships among the three genera were also well resolved (Fig. 4). In our maximum likelihood (ML) phylogeny, the genus *Neotessella* is the deepest branching synurophycean lineage, with *Mallomonas* and *Synura* splitting off thereafter. Furthermore, our phylogenomic investigations show that the Synurophyceae form a strongly supported sister relationship with the chrysophytes (Fig. 4), which is congruent with previous multigene phylogenetic studies [9, 10, 40–42]. Interestingly, the chromerids *V. brassicaformis* and *C. velia* form a strongly supported sister relationship with Eustigmatophyceae in Fig. 4. This topology is consistent with recent studies suggesting that the eustigmatophytes could be the source of the chromerid plastid [5, 43].

Synurophycean algae are characterized by the presence of distinctive siliceous scales that produce a highly organized covering around the cell [8, 44]. The fossil record is rich in Synurophyceae containing silicious scales and cysts, which are resting stages produced by species of the Synurophyceae as well as Chrysophyceae [8, 45]. According to Siver et al. [8], the Synurophyceae originated in the Jurassic, approximately 157 million years ago (Ma), with the clade containing *Mallomonas* and *Synura* diverging during the Early Cretaceous at 130 Ma.

**Fig. 5:**
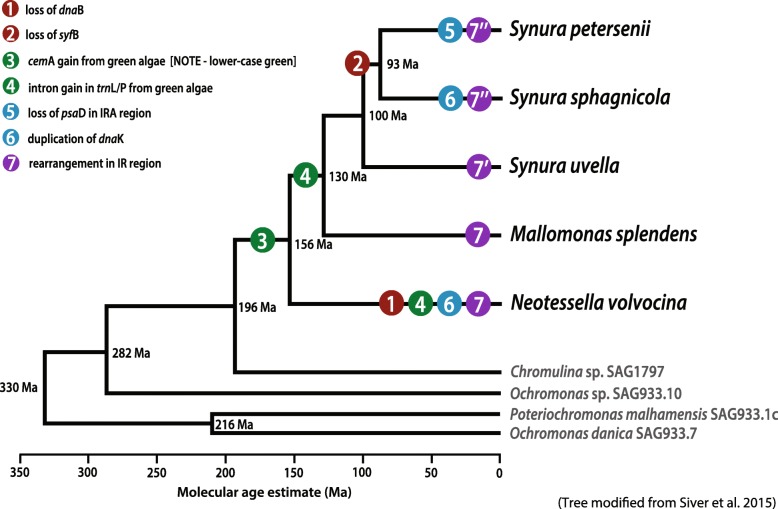
Molecular timeline of synurophyte plastid genomes. Putative gene loss, LGT, and intron insertion events are mapped onto a schematic tree modified from the time-calibrated multi-gene phylogeny of Siver et al. [8]. The number at each node represents the mean divergence time (in millions of years)

Using molecular clock data and our plastid genome phylogenies, we inferred the timing of gene gains, losses, and rearrangements in the plastid genomes of the synurophycean lineage. *N. volvocina* is predicted to have lost the *dna*B gene in the plastid genome between ~ 156 Ma and the present, after the major synurophycean lineages diverged (Fig. 5. ①). The *syf*B gene loss may have occurred during the Early Cretaceous at 130 Ma, after the divergence of colonial *Synura* and unicellular *Mallomonas*; this is inferred because the gene is found in the plastid genomes of *Mallomonas, Synura, Neotessela* and ochrophytes (Fig. 5. ②). The *cem*A gene, hypothesized to have been derived from a member of the Viridiplantae by LGT, and the intron of the *trn*L^UAA^ (*Synura* and *Mallomonas*) or *trn*P^UGG^ (*N. volvocina*) genes appear to have been acquired during the Jurassic approximately 156 Ma, before the divergence of the *Mallomonas*, *Neotessela*, and *Synura* genera (Fig. 5. ③-④). The *psa*D gene, located near *dna*K in the LSC/IRA junction, appears to have been lost ~ 93 Ma before present because the gene is found in all genera except *S. petersenii* (Fig. 5. ⑤). The partial duplication of the *dna*K gene near the *ilv*B gene in the SSC/IRB junction might have been duplicated or truncated recently given that the genes are present only in *S. uvella* and *N. volvocina* (Fig. 5. ⑤-⑥). The gene rearrangements in the IR regions and duplications/translocations in the SSC regions are the result of species-specific events (Fig. 5. ⑦).

## Conclusions

We have sequenced five synurophyte plastid genomes from morphologically distinct genera: the colonial genus *Synura*, whose individual cells are covered with silica scales; the single-celled genus *Mallomonas* covered with silica scales and bristles; and the colonial genus *Neotessella*, whose entire colony is covered with a single, large silica case. The overall organization of the plastid genome shows a high degree of conservation among the five Synurophyceae and *Ochromonas* sp. CCMP1393, but *N. volvocina* has two inversions relative to the other synurophycean species. The IR and SSC boundaries are particularly variable from species to species. Instances of lineage specific gene loss/gain and intron insertions were also detected (e.g., *cem*A, *dna*B, *syf*B, and *trn*L). The *dna*B and *syf*B genes appear to have been lost independently in different synurophyceans. Both the *trn*L intron sequences and *cem*A gene of Synurophyceae appear most closely related to their counterparts in green algae, suggestive of LGT. However, their sequences are divergent and should thus be interpreted with caution. All things considered, the extent to which LGT has contributed to the plastid genomes of Synurophyceae and other algae remains to be seen. Multi-gene phylogenetic analyses show that Synurophyceae group together with Chrysophyceae among the stramenopiles. Combined with molecular clock data, our phylogenetic tree allows us to infer the timing of gene gains, losses, duplications and rearrangements in the plastid genome of the synurophycean lineage.

## Materials and methods

### Cultures and sequencing

Cultures of *Neotessella volvocina* CCMP1871 and *Mallomonas splendens* CCMP1872 were obtained from the Culture Collection of the National Center for Marine Algae and Microbiota (NCMA). Three species of *Synura* were collected from natural habitats: *Synura petersenii* from Sweden (36° 30′ N, 126° 47′ E), *Synura sphagnicola* from Cheongyang, Korea (36° 30′ N, 126° 47′ E), and *Synura uvella* from Gahang, Korea (35° 30′ N, 128° 23′ E). *S. petersenii* strain S114.C7 has been deposited as CAUP B713 in the Culture Collection of Algae of Charles University in Prague, Czech Republic. The strains *S. sphagnicola* FBCC200022 and *S. uvella* FBCC200023 are available from the Freshwater Bioresources Culture Collection at the Nakdong-gang National Institute of Biological Resources Korea, respectively. All freshwater cultures were grown in DY-V medium [46] with distilled water and were maintained at 17 °C under conditions of a 14:10 light:dark cycle with 30 μmol photons·m^− 2^·s^− 1^ from cool white fluorescent tubes. All cultures were derived from a single-cell isolate for unialgal cultivation. Total genomic DNAs were extracted using the QIAGEN DNEasy Blood Mini Kit (QIAGEN, Valencia, CA, USA) following the manufacturer’s instructions. Next-generation sequencing was carried out using the MiSeq (Illumina, San Diego, CA, USA). The amplified DNA was fragmented and tagged using the NexteraXT protocol (Illumina), indexed, size selected, and pooled for sequencing using the small amplicon targeted resequencing run, which performs paired end 2 × 300 bp sequencing reads using the MiSeq Reagent Kit v3 (Illumina), according to the manufacturer’s recommendations.

### Assembly and annotation of plastid genomes

Sequence data were trimmed, assembled using the SPAdes 3.7 assembler (http://bioinf.spbau.ru/spades), and mapped to the assembled contigs. The contigs were deemed to be of plastid genome origin as follows: (1) BLAST searches against the entire assembly using commonly known plastid genes as queries resulted in hits to these contigs [47, 48] and (2) the predicted genome sizes were similar to the previously published 127 kbp plastid genome of *Ochromonas* sp. CCMP1393 (KJ877675). For each genome we verified the sequence and structure of both inverted repeat positions and SSC regions with specific primers using standard Sanger sequencing (Additional file 5: Figure S1). Annotation of protein coding genes, rRNA genes, and tRNA genes were identified using data from all previously sequenced synurophycean plastid genomes according to the methods described in Kim et al. [16]. Genome sequences were deposited to the NCBI GenBank database under the accession numbers shown in Table 1.

### Phylogenetic analysis

Phylogenetic analyses were carried out on amino acid sequence datasets created by combining 91 protein coding genes from 99 plastid genomes (Additional file 6: Table S1). The sequences of six Viridiplantae and one glaucophyte species were used as outgroup taxa for rooting purposes. The datasets were aligned and concatenated (18,250 amino acid sequences) using MacGDE2.6 [49].

ML phylogenetic analyses of individual protein alignments and concatenated alignments were conducted using IQ-TREE Ver. 1.5.2 [50] with 1000 bootstrap replications. The best evolutionary model for each tree was automatically selected using the –m LG + I + G option incorporated in IQ-TREE. RAxML version 8.0.0 [51] with the general time-reversible plus gamma (GTR + GAMMA) model was used for nucleotide data of the intron within *trn*L^UAA^. The model parameters with gamma correction values and the proportion of invariable sites in the combined dataset were obtained automatically by the program. ML bootstrap support values (MLB) were calculated using 1000 replicates with the same substitution model. Bayesian analyses were carried out using MrBayes 3.2.6 [52] with two simultaneous runs (nruns = 2). Four Metropolis-coupled Markov chain Monte Carlo (MC^3^) chains ran for 2 × 10^6^ generations, sampling every 1000 generations. The burn-in point was determined by examining the trace files using Tracer v.1.6 (http://tree.bio.ed.ac.uk/software/tracer/). This analysis was repeated twice independently, and both analyses resulted in the same tree. The trees were visualized using the FigTree v.1.4.2 program, available at http://tree.bio.ed.ac.uk/software/figtree/.

## Additional files

Additional file 1: **Figure S2.** Overview of Synurophyceae plastid genomes. Linearized maps of five novel complete plastid genomes are compared with *Ochromonas* sp. CCMP1393. The color coded syntenic blocks are shown above each genome, and the gene maps are shown below each genome. The syntenic blocks above the horizontal line are on the same strand, and those below the line are on the opposite strand. The horizontal bars inside the syntenic blocks show sequence conservation. The block boundaries correspond to the sites where inversion events occurred. In the gene maps, the genes above the horizontal line are transcribed from left to right, and those below the horizontal line are transcribed from right to left. The rRNA operons are shown in red. (PDF 1586 kb)

Additional file 2: **Figure S3.** Phylogenetic tree based on *dna*B. Numbers on branches are IQ-Tree UFBoot values. The scale bar shows the inferred number of amino acid substitutions per site. (PDF 283 kb)

Additional file 3: **Figure S4.** Phylogenetic tree based on *syf*B. Numbers on branches are IQ-Tree UFBoot values. The scale bar shows the inferred number of amino acid substitutions per site. (PDF 227 kb)

Additional file 4: **Figure S5.** Phylogenetic tree based on intron sequence within *trn*L^UAA^. Numbers on branches are RAxML bootstrap values. The scale bar indicates the number of substitutions/site. (PDF 178 kb)

Additional file 5: **Figure S1.** The positions of gap filling with primer information. (PDF 489 kb)

Additional file 6: **Table S1.** The concatenated dataset of protein sequences used to infer the reference phylogenetic tree. (XLSX 53 kb)

## Abbreviations

CER: Chloroplast ER
IR: Inverted repeats
LGT: Lateral gene transfer
LSC: Large single copy region
Ma: Milion years ago
ML: Maximum likelihood
MLB: Maximum likelihood bootstrap
ORF: Open reading frame
PheRS: Phenylalanyl-tRNA synthetase
ptDNA: Plastid genomes
SDVs: Silica deposition vesicles
SSC: Short single copy region
