# Supplementary material for: Comparative plastid genomics of Synurophyceae: inverted repeat dynamics and gene content variation
Source: BMC Evol Biol. 2019 Jan 11;19:20. doi: 10.1186/s12862-018-1316-9 (PMC6330437; doi:10.1186/s12862-018-1316-9)
Supplement: Supplementary file 1 — Figure S2. Overview of Synurophyceae plastid genomes. Linearized maps of five novel complete plastid genomes are compared with Ochromonas sp. CCMP1393. The color coded syntenic blocks are shown above each genome, and the gene maps are shown below each genome. The syntenic blocks above the horizontal line are on the same strand, and those below the line are on the opposite strand. The horizontal bars inside the syntenic blocks show sequence conservation. The block boundaries correspond to the sites where inversion events occurred. In the gene maps, the genes above the horizontal line are transcribed from left to right, and those below the horizontal line are transcribed from right to left. The rRNA operons are shown in red. (PDF 1586 kb) [file 12862_2018_1316_MOESM1_ESM.pdf]

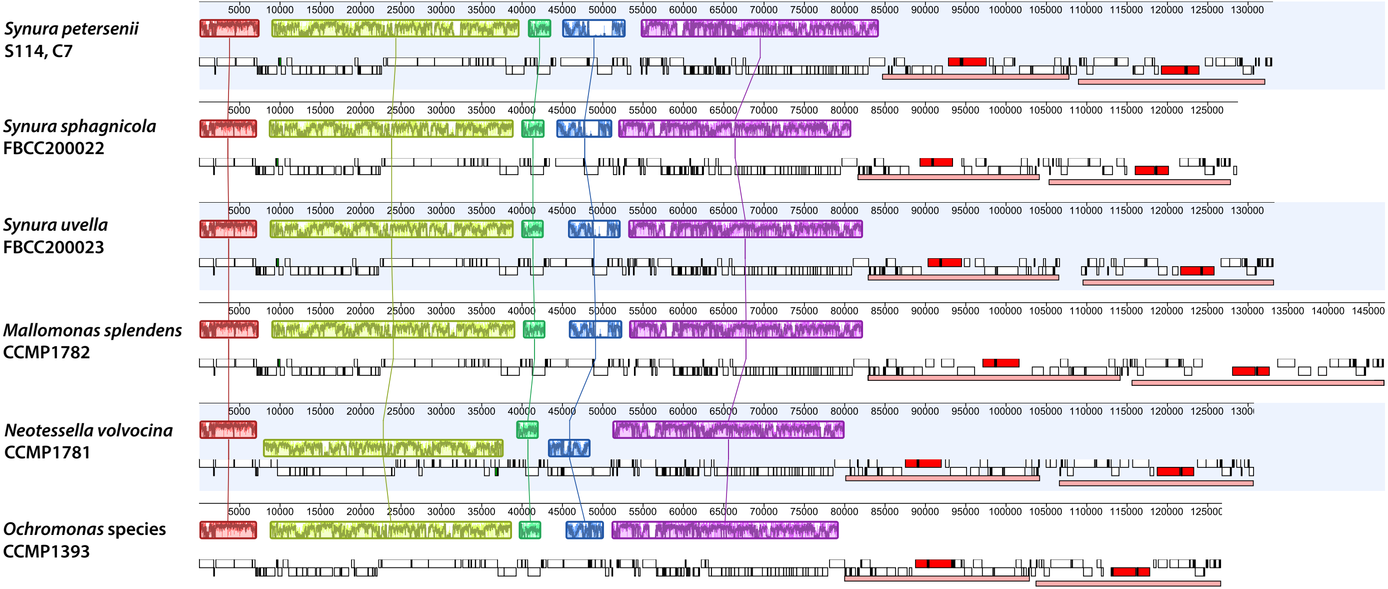

Synura petersenii S114, C7
5000 10000 15000 20000 25000 30000 35000 40000 45000 50000 55000 60000 65000 70000 75000 80000 85000 90000 95000 100000 105000 110000 115000 120000 125000 130000
Synura sphagnicola FBCC200022
5000 10000 15000 20000 25000 30000 35000 40000 45000 50000 55000 60000 65000 70000 75000 80000 85000 90000 95000 100000 105000 110000 115000 120000 125000
Synura uvella FBCC200023
5000 10000 15000 20000 25000 30000 35000 40000 45000 50000 55000 60000 65000 70000 75000 80000 85000 90000 95000 100000 105000 110000 115000 120000 125000 130000
Mallomonas splendens CCMP1782
5000 10000 15000 20000 25000 30000 35000 40000 45000 50000 55000 60000 65000 70000 75000 80000 85000 90000 95000 100000 105000 110000 115000 120000 125000 130000 135000 140000 145000
Neotessella volvocina CCMP1781
5000 10000 15000 20000 25000 30000 35000 40000 45000 50000 55000 60000 65000 70000 75000 80000 85000 90000 95000 100000 105000 110000 115000 120000 125000
Ochromonas species CCMP1393
5000 10000 15000 20000 25000 30000 35000 40000 45000 50000 55000 60000 65000 70000 75000 80000 85000 90000 95000 100000 105000 110000 115000 120000
