# Supplementary material for: Comparative plastid genomics of Synurophyceae: inverted repeat dynamics and gene content variation
Source: BMC Evol Biol. 2019 Jan 11;19:20. doi: 10.1186/s12862-018-1316-9 (PMC6330437; doi:10.1186/s12862-018-1316-9)
Supplement: Supplementary file 2 — Figure S3. Phylogenetic tree based on dnaB. Numbers on branches are IQ-Tree UFBoot values. The scale bar shows the inferred number of amino acid substitutions per site. (PDF 283 kb) [file 12862_2018_1316_MOESM2_ESM.pdf]

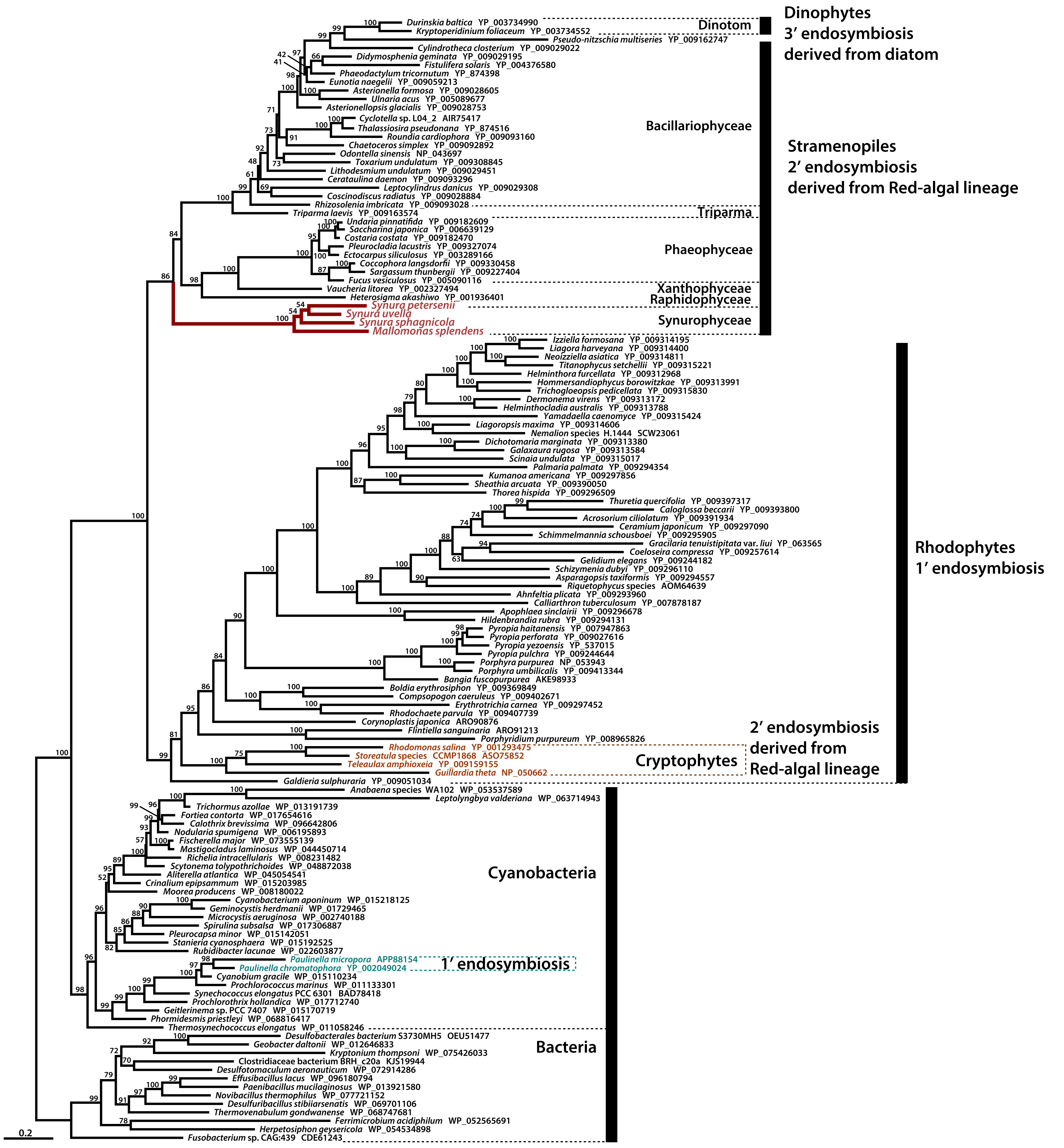
Dinophytes
3′ endosymbiosis
derived from diatom
Dinotom
Durinskia baltica YP_003734990
Kryptoperidinium foliaceum YP_003734552
Pseudo-nitzschia multiseries YP_009162747
Cylindrotheca closterium YP_009029022
Didymosphenia geminata YP_009029195
Fistulifera solaris YP_004376580
Phaeodactylum tricornutum YP_874398
Eunotia naegelii YP_009059213
Asterionella formosa YP_009028605
Ulnaria acus YP_005089677
Asterionellopsis glacialis YP_009028753
Cyclotella sp. L04_2 AIR75417
Thalassiosira pseudonana YP_874516
Roundia cardiophora YP_009093160
Chaetoceros simplex YP_009092892
Odontella sinensis NP_043697
Toxarium undulatum YP_009308845
Lithodesmium undulatum YP_009029451
Cerataulina daemon YP_009093296
Leptocylindrus danicus YP_009029308
Coscinodiscus radiatus YP_009028884
Rhizosolenia imbricata YP_009093028
Triparma laevis YP_009163574
Bacillariophyceae
Triparma
Stramenopiles
2′ endosymbiosis
derived from Red-algal lineage
Undaria pinnatifida YP_009182609
Saccharina japonica YP_006639129
Costaria costata YP_009182470
Pleurocladia lacustris YP_009327074
Ectocarpus siliculosus YP_003289166
Coccophora langsdorfii YP_009330458
Sargassum thunbergii YP_009227404
Fucus vesiculosus YP_005090116
Vaucheria litorea YP_002327494
Heterosigma akashiwo YP_001936401
Phaeophyceae
Xanthophyceae
Raphidophyceae
Synura petersenii
Synura uvella
Synura sphagnicola
Mallomonas splendens
Synurophyceae
Izziella formosana YP_009314195
Liagora harveyana YP_009314400
Neoizziella asiatica YP_009314811
Titanophycus setchellii YP_009315221
Helminthora furcellata YP_009312968
Hommersandiophycus borowitzkae YP_009313991
Trichogloeopsis pedicellata YP_009315830
Dermonema virens YP_009313172
Helminthocladia australis YP_009313788
Yamadaella caenomyce YP_009315424
Liagoropsis maxima YP_009314606
Nemalion species H.1444 SCW23061
Dichotomaria marginata YP_009313380
Galaxaura rugosa YP_009313584
Scinaia undulata YP_009315017
Palmaria palmata YP_009294354
Kumanoa americana YP_009297856
Sheathia arcuata YP_009390050
Thorea hispida YP_009296509
Thuretia quercifolia YP_009397317
Caloglossa beccarii YP_009393800
Acrosorium ciliolatum YP_009391934
Ceramium japonicum YP_009297090
Schimmelmannia schousboei YP_009295905
Gracilaria tenuistipitata var. liui YP_063565
Coeloseira compressa YP_009257614
Gelidium elegans YP_009244182
Schizymenia dubyi YP_009296110
Asparagopsis taxiformis YP_009294557
Riquetophycus species AOM64639
Ahnfeltia plicata YP_009293960
Calliarthron tuberculosum YP_007878187
Apophlaea sinclairii YP_009296678
Hildenbrandia rubra YP_009294131
Pyropia haitanensis YP_007947863
Pyropia perforata YP_009027616
Pyropia yezoensis YP_537015
Pyropia pulchra YP_009244644
Porphyra purpurea NP_053943
Porphyra umbilicalis YP_009413344
Bangia fuscopurpurea AKE98933
Boldia erythrosiphon YP_009369849
Compsopogon caeruleus YP_009402671
Erythrotrichia carnea YP_009297452
Rhodochaete parvula YP_009407739
Corynoplastis japonica ARO90876
Flintiella sanguinaria ARO91213
Porphyridium purpureum YP_008965826
Rhodomonas salina YP_001293475
Storeatula species CCMP1868 ASO75852
Teleaulax amphioxeia YP_009159155
Guillardia theta NP_050662
Galdieria sulphuraria YP_009051034
Rhodophytes
1′ endosymbiosis
2′ endosymbiosis
derived from
Red-algal lineage
Cryptophytes
Anabaena species WA102 WP_053537589
Leptolyngbya valderiana WP_063714943
Trichormus azollae WP_013191739
Fortiea contorta WP_017654616
Calothrix brevissima WP_096642806
Nodularia spumigena WP_006195893
Fischerella major WP_073555139
Mastigocladus laminosus WP_044450714
Richelia intracellularis WP_008231482
Scytonema tolypothrichoides WP_048872038
Aliterella atlantica WP_045054541
Crinalium epipsammum WP_015203985
Moorea producens WP_008180022
Cyanobacterium aponinum WP_015218125
Geminocystis herdmanii WP_01729465
Microcystis aeruginosa WP_002740188
Spirulina subsalsa WP_017306887
Pleurocapsa minor WP_015142051
Stanieria cyanosphaera WP_015192525
Rubidibacter lacunae WP_022603877
Paulinella micropora APP88154
Paulinella chromatophora YP_002049024
Cyanobium gracile WP_015110234
Prochlorococcus marinus WP_011133301
Synechococcus elongatus PCC 6301 BAD78418
Prochlorothrix hollandica WP_017712740
Geitlerinema sp. PCC 7407 WP_015170719
Phormidesmis priestleyi WP_068816417
Thermosynechococcus elongatus WP_011058246
Cyanobacteria
1′ endosymbiosis
Desulfobacterales bacterium S3730MH5 OEU51477
Geobacter daltonii WP_012646833
Kryptonium thompsoni WP_075426033
Clostridiaceae bacterium BRH_c20a KJS19944
Desulfotomaculum aeronauticum WP_072914286
Effusibacillus lacus WP_096180794
Paenibacillus mucilaginosus WP_013921580
Novibacillus thermophilus WP_077721152
Desulfuribacillus stibiiarsenatis WP_069701106
Thermovenabulum gondwanense WP_068747681
Ferrimicrobium acidiphilum WP_052565691
Herpetosiphon geysericola WP_054534898
Fusobacterium sp. CAG:439 CDE61243
Bacteria
0.2
