# Supplementary material for: Comparative plastid genomics of Synurophyceae: inverted repeat dynamics and gene content variation
Source: BMC Evol Biol. 2019 Jan 11;19:20. doi: 10.1186/s12862-018-1316-9 (PMC6330437; doi:10.1186/s12862-018-1316-9)
Supplement: Supplementary file 3 — Figure S4. Phylogenetic tree based on syfB. Numbers on branches are IQ-Tree UFBoot values. The scale bar shows the inferred number of amino acid substitutions per site. (PDF 227 kb) [file 12862_2018_1316_MOESM3_ESM.pdf]

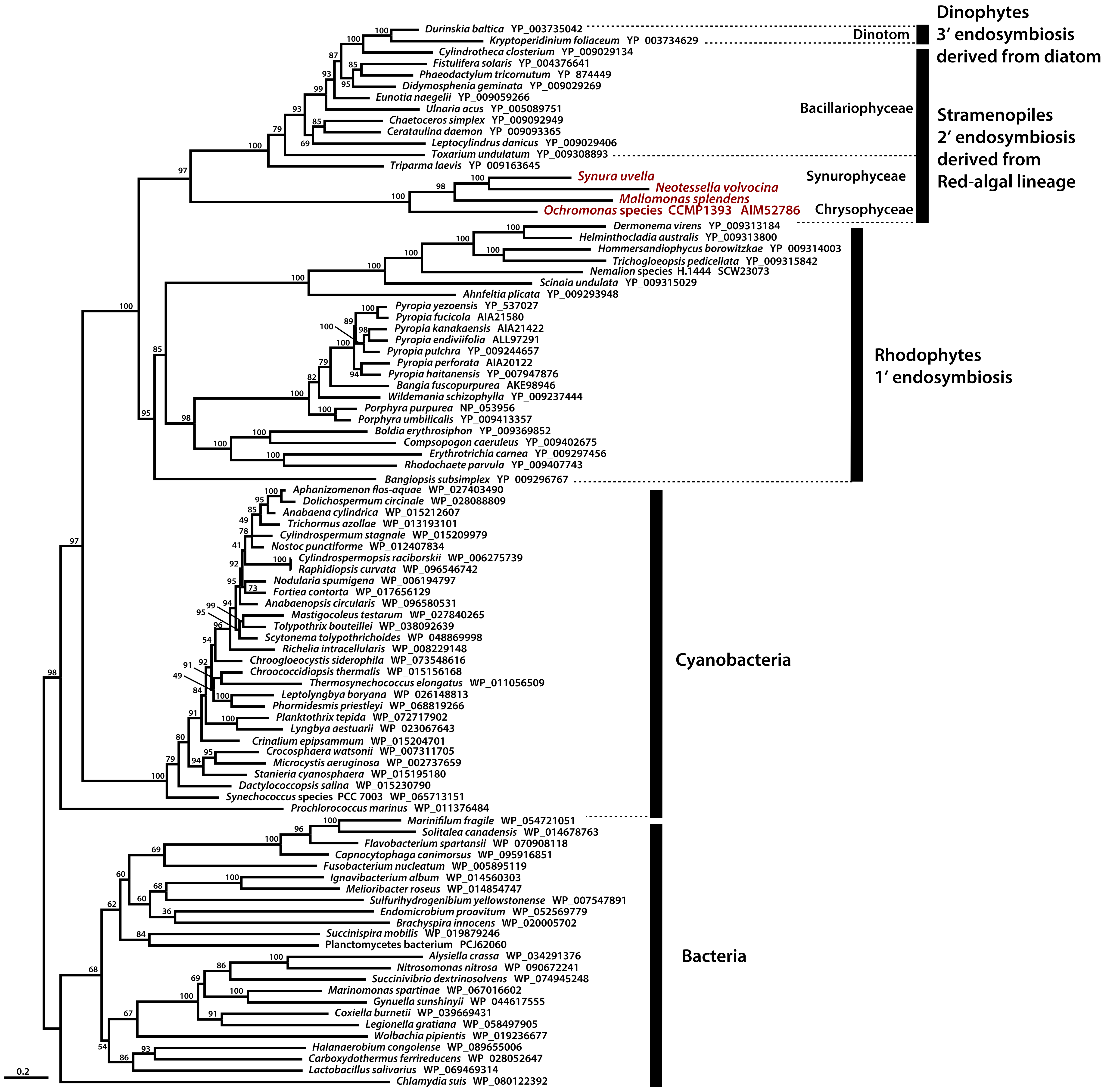
Dinophytes
3′ endosymbiosis
derived from diatom
Dinotom
Stramenopiles
2′ endosymbiosis
derived from
Red-algal lineage
Bacillariophyceae
Synurophyceae
Chrysophyceae
Rhodophytes
1′ endosymbiosis
Cyanobacteria
Bacteria
Durinskia baltica YP_003735042
Kryptoperidinium foliaceum YP_003734629
Cylindrotheca closterium YP_009029134
Fistulifera solaris YP_004376641
Phaeodactylum tricornutum YP_874449
Didymosphenia geminata YP_009029269
Eunotia naegelii YP_009059266
Ulnaria acus YP_005089751
Chaetoceros simplex YP_009092949
Cerataulina daemon YP_009093365
Leptocylindrus danicus YP_009029406
Toxarium undulatum YP_009308893
Triparma laevis YP_009163645
Synura uvella
Neotessella volvocina
Mallomonas splendens
Ochromonas species CCMP1393 AIM52786
Dermonema virens YP_009313184
Helminthocladia australis YP_009313800
Hommersandiophycus borowitzkae YP_009314003
Trichogloeopsis pedicellata YP_009315842
Nemalion species H.1444 SCW23073
Scinaia undulata YP_009315029
Ahnfeltia plicata YP_009293948
Pyropia yezoensis YP_537027
Pyropia fucicola AIA21580
Pyropia kanakaensis AIA21422
Pyropia endiviifolia ALL97291
Pyropia pulchra YP_009244657
Pyropia perforata AIA20122
Pyropia haitanensis YP_007947876
Bangia fuscopurpurea AKE98946
Wildemania schizophylla YP_009237444
Porphyra purpurea NP_053956
Porphyra umbilicalis YP_009413357
Boldia erythrosiphon YP_009369852
Compsopogon caeruleus YP_009402675
Erythrotrichia carnea YP_009297456
Rhodochaete parvula YP_009407743
Bangiopsis subsimplex YP_009296767
Aphanizomenon flos-aquae WP_027403490
Dolichospermum circinale WP_028088809
Anabaena cylindrica WP_015212607
Trichormus azollae WP_013193101
Cylindrospermum stagnale WP_015209979
Nostoc punctiforme WP_012407834
Cylindrospermopsis raciborskii WP_006275739
Raphidiopsis curvata WP_096546742
Nodularia spumigena WP_006194797
Fortiea contorta WP_017656129
Anabaenopsis circularis WP_096580531
Mastigocoleus testarum WP_027840265
Tolypothrix bouteillei WP_038092639
Scytonema tolypothrichoides WP_048869998
Richelia intracellularis WP_008229148
Chroogloeocystis siderophila WP_073548616
Chroococcidiopsis thermalis WP_015156168
Thermosynechococcus elongatus WP_011056509
Leptolyngbya boryana WP_026148813
Phormidesmis priestleyi WP_068819266
Planktothrix tepida WP_072717902
Lyngbya aestuarii WP_023067643
Crinalium epipsammum WP_015204701
Crocosphaera watsonii WP_007311705
Microcystis aeruginosa WP_002737659
Stanieria cyanosphaera WP_015195180
Dactylococcopsis salina WP_015230790
Synechococcus species PCC 7003 WP_065713151
Prochlorococcus marinus WP_011376484
Marinifilum fragile WP_054721051
Solitalea canadensis WP_014678763
Flavobacterium spartansii WP_070908118
Capnocytophaga canimorsus WP_095916851
Fusobacterium nucleatum WP_005895119
Ignavibacterium album WP_014560303
Melioribacter roseus WP_014854747
Sulfurihydrogenibium yellowstonense WP_007547891
Endomicrobium proavitum WP_052569779
Brachyspira innocens WP_020005702
Succinispira mobilis WP_019879246
Planctomycetes bacterium PCJ62060
Alysiella crassa WP_034291376
Nitrosomonas nitrosa WP_090672241
Succinivibrio dextrinosolvens WP_074945248
Marinomonas spartinae WP_067016602
Gynuella sunshinyii WP_044617555
Coxiella burnetii WP_039669431
Legionella gratiana WP_058497905
Wolbachia pipientis WP_019236677
Halanaerobium congolense WP_089655006
Carboxydothermus ferrireducens WP_028052647
Lactobacillus salivarius WP_069469314
Chlamydia suis WP_080122392
0.2
