# Supplementary figures and images for: Comparative plastid genomics of Synurophyceae: inverted repeat dynamics and gene content variation
Source: BMC Evol Biol. 2019 Jan 11;19:20. doi: 10.1186/s12862-018-1316-9 (PMC6330437; doi:10.1186/s12862-018-1316-9)

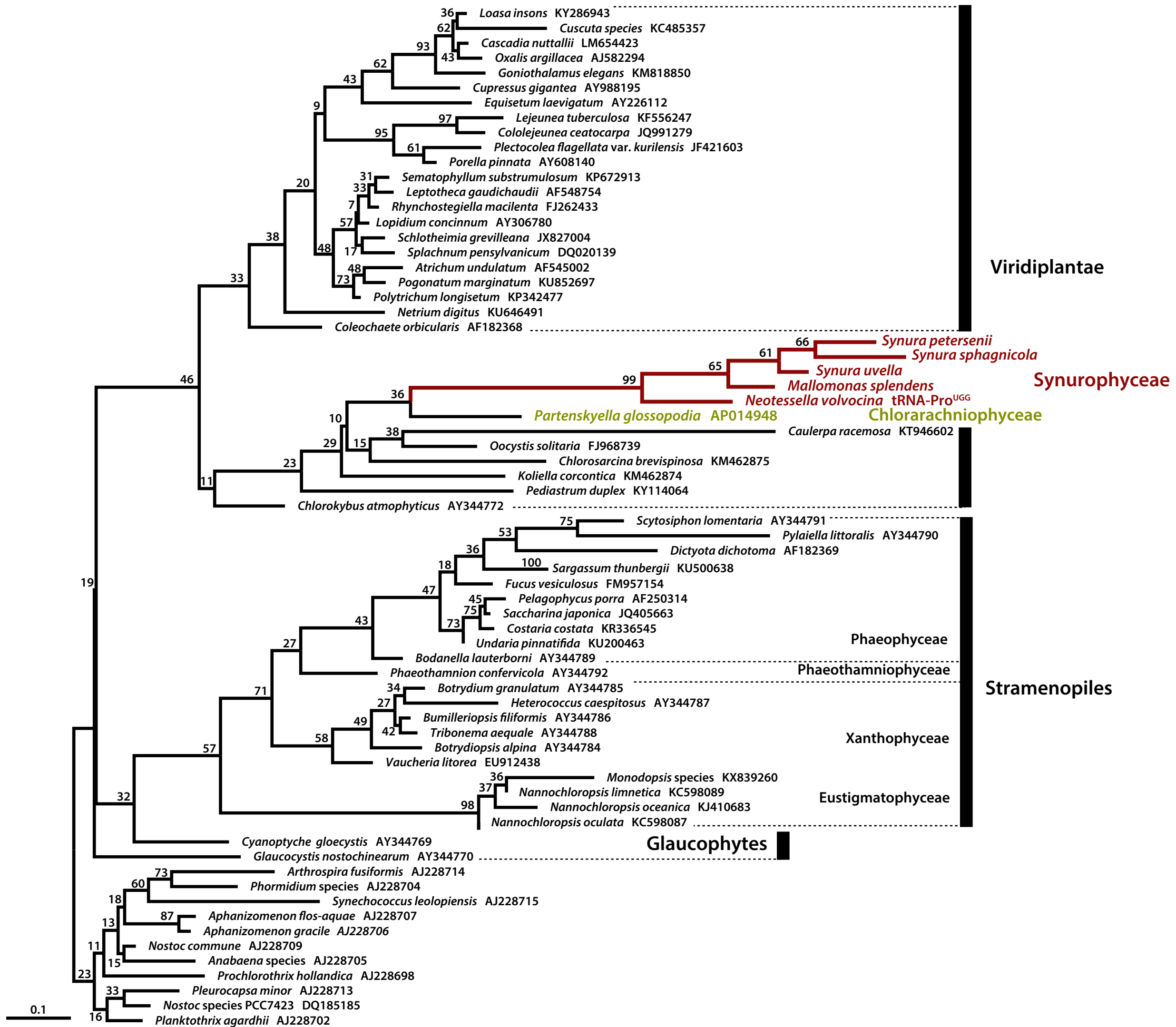

Supplement: Supplementary file 4 — Figure S5. Phylogenetic tree based on intron sequence within trnLUAA. Numbers on branches are RAxML bootstrap values. The scale bar indicates the number of substitutions/site. (PDF 178 kb) [file 12862_2018_1316_MOESM4_ESM.pdf]

# The Primer Sequences for Gap Filling

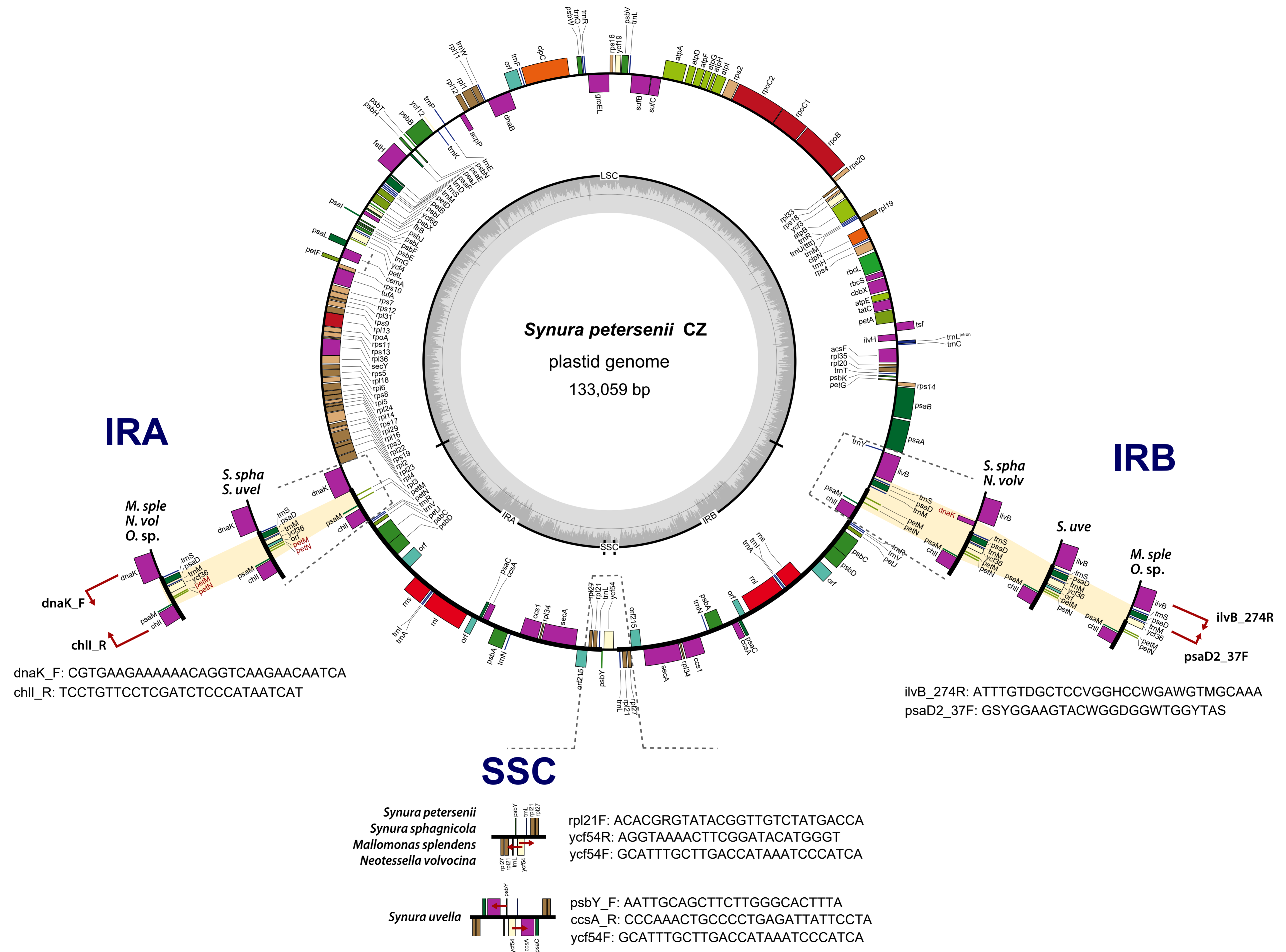

Supplement: Supplementary file 5 — Figure S1. The positions of gap filling with primer information. (PDF 489 kb) [file 12862_2018_1316_MOESM5_ESM.pdf]
